# A regulatory role for CHD2 in myelopoiesis

**DOI:** 10.1080/15592294.2019.1710913

**Published:** 2020-01-10

**Authors:** Farzaneh Shahin Varnoosfaderani, Anna Palau, Wenbo Dong, Jenna Persson, Mickaël Durand-Dubief, J Peter Svensson, Andreas Lennartsson

**Affiliations:** a Department of Biosciences and Nutrition, Neo, Karolinska Institutet, Stockholm, Sweden; b Department of Medical Biochemistry and Biophysics, Karolinska Institutet, Stockholm, Sweden; c High Throughput Genome Engineering, Science for Life Laboratory, Stockholm, Sweden

**Keywords:** CHD2, myelopoiesis, CRISPR-Cas9 library, epigenetics

## Abstract

The transcriptional program that dictates haematopoietic cell fate and differentiation requires an epigenetic regulatory and memory function, provided by a network of epigenetic factors that regulate DNA methylation, post-translational histone modifications and chromatin structure. Disturbed epigenetic regulation causes perturbations in the blood cell differentiation program that results in various types of haematopoietic disorders. Thus, accurate epigenetic regulation is essential for functional haematopoiesis. In this study, we used a CRISPR-Cas9 screening approach to identify new epigenetic regulators in myeloid differentiation. We designed a Chromatin-UMI CRISPR guide library targeting 1092 epigenetic regulators. Phorbol 12-myristate 13-acetate (PMA) treatment of the chronic myeloid leukaemia cell line K-562 was used as a megakaryocytic myeloid differentiation model. Both previously described developmental epigenetic regulators and novel factors were identified in our screen. In this study, we validated and characterized a role for the chromatin remodeller CHD2 in myeloid proliferation and megakaryocytic differentiation.

## Introduction

An interactive network of transcription factors and epigenetic regulators tightly regulates haematopoiesis [1]. Timed, cell-specific expression of epigenetic regulators provides a framework for cell-specific chromatin structure [2]. DNA methylation has been shown to decrease with myeloid differentiation, especially in enhancer regions [3]. Histone modifications are also essential for normal myelopoiesis. The Polycomb repressive complexes 1 and 2 that regulate H2A lysine 119 monoubiquitylation and H3 lysine 27 trimethylation respectively, are required for haematopoietic stem cell (HSC) function and differentiation [4,5]. In addition, the importance of epigenetic regulation in myelopoiesis is demonstrated by the high frequency of mutations and chromosomal translocations of epigenetic enzymes in myeloproliferative and myelodysplastic disorders and in the development of Acute Myeloid Leukaemia (AML) [6]. AML is the most common acute leukaemia in adults and still has a very bad prognosis, with only approximately 25–30% long-term survival. Mutations in epigenetic regulation factors, such as *DNMT3A* and *TET2*, are present in preleukemic haematopoietic stem cells and occurs early in the evolution of AML [7]. Most likely, mutations of epigenetic regulators increase epigenetic instability, which can lead to more mutations causing leukaemia [6]. The alterations in epigenetic regulators affect DNA methylation, histone modifications and chromatin regulatory mechanisms [6].

While the functions of DNA methylation and some histone modifications in myelopoiesis have been well described [1], the role of chromatin remodelling factors and complexes is still not determined. ATP-dependent chromatin remodellers regulate nucleosome mobilization, such as assembly/disassembly, eviction, sliding and spacing of nucleosomes [8]. The chromodomain helicase DNA-binding protein (CHD) family of chromatin remodellers has been shown to play an important role in stem cell function, differentiation and cancer [9–11]. CHD1 has previously been demonstrated to be important for pluripotency and CHD4 was recently described to be required for maintenance of childhood AML [10,12], suggesting a crucial role for the CHD-family in haematopoiesis.

In this study, we used CRISPR-Cas9 screening to identify novel epigenetic regulators that control myeloid differentiation. We identified a novel role for the CHD2 chromatin remodeller in regulating proliferation and megakaryocytic differentiation.

## Results

### Identification of epigenetic factors that regulate myeloid differentiation

**Figure 1.**
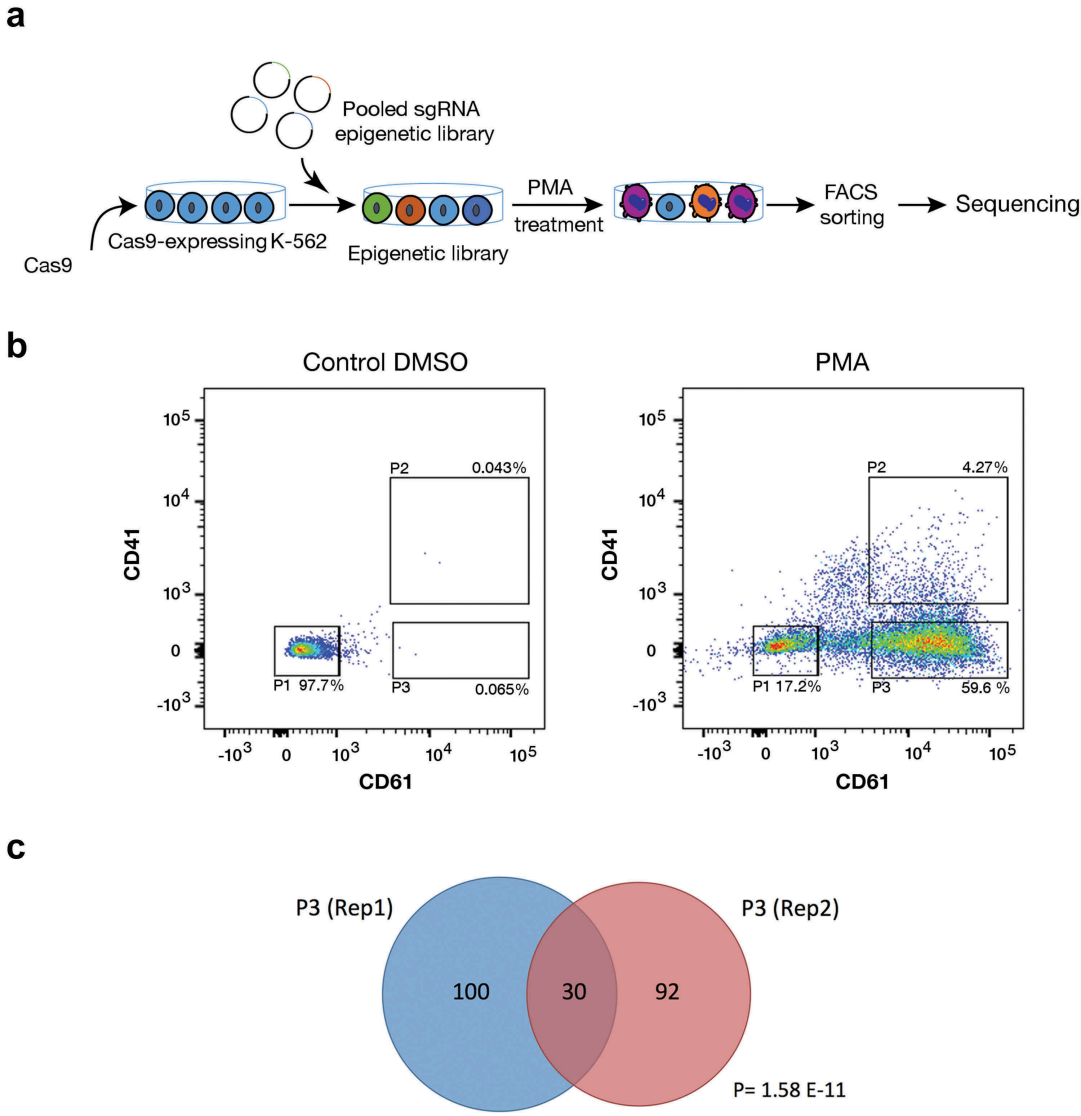
CRISPR-Cas9 screening for epigenetic factors. (a) Schematic overview for CRISPR-Cas9 screen in K-562 cells. Cas-9 expressing K-562 cells were transduced with chromatin-UMI library. Cells were treated with 5 nM PMA for 72 h, sorted and sequenced (n = 2). (b) Representative Flow cytometry chart for CRISPR-Cas9-K-562 cells after 5 nM PMA treatment for 72 h. Treated cells were sorted for CD61 and CD41 with three different gatings: P1 (CD61/CD41−), P2 (CD61/CD41++), and P3 (CD61+). (c) Venn diagram graph shows significant (Hypogeometric distribution P = 1.58 E-11) overlap of enriched sgRNAs between two biological replicates of the CRISPR-Cas9 screens.

Differentiation of the chronic myeloid leukaemic cell line K-562 to the megakaryocytic lineage with Phorbol 12-myristate 13-acetate (PMA) was used as a model system for myeloid maturation. PMA treatment induces megakaryocytic differentiation and expression of the megakaryocytic markers CD61 and CD41 [13]. A K-562 cell line that stably expresses CRISPR-Cas9 was established and transduced with a Unique Molecular Identifier (UMI) CRISPR guide library [14] that targets 1092 epigenetic regulators and 120 control genes with four sgRNAs each, plus 200 non-targeting guides (Figure 1(a)). The UMIs are used to track each transduction event. The transduced cells were treated with either PMA or DMSO for 72 h. Megakaryocytic differentiation was analysed both by morphology (Figure S1) and by flow cytometry through the expression of the surface markers CD41 and CD61. To identify epigenetic regulators that affect differentiation, three different cell populations (n = 2) were sorted and collected by FACS: population P1 that consists of undifferentiated cells (CD61− and CD41−) and two populations with at least one megakaryocytic differentiation marker present: P2 (CD61+ and CD41+) and P3 (CD61+ and CD41−) (Figure 1(b)). Genomic DNA from each sorted cell population and from unsorted cells was extracted, and guide and UMI sequences were amplified by PCR and sequenced by Next Generation Sequencing (NGS) [14].

**Figure 2.**
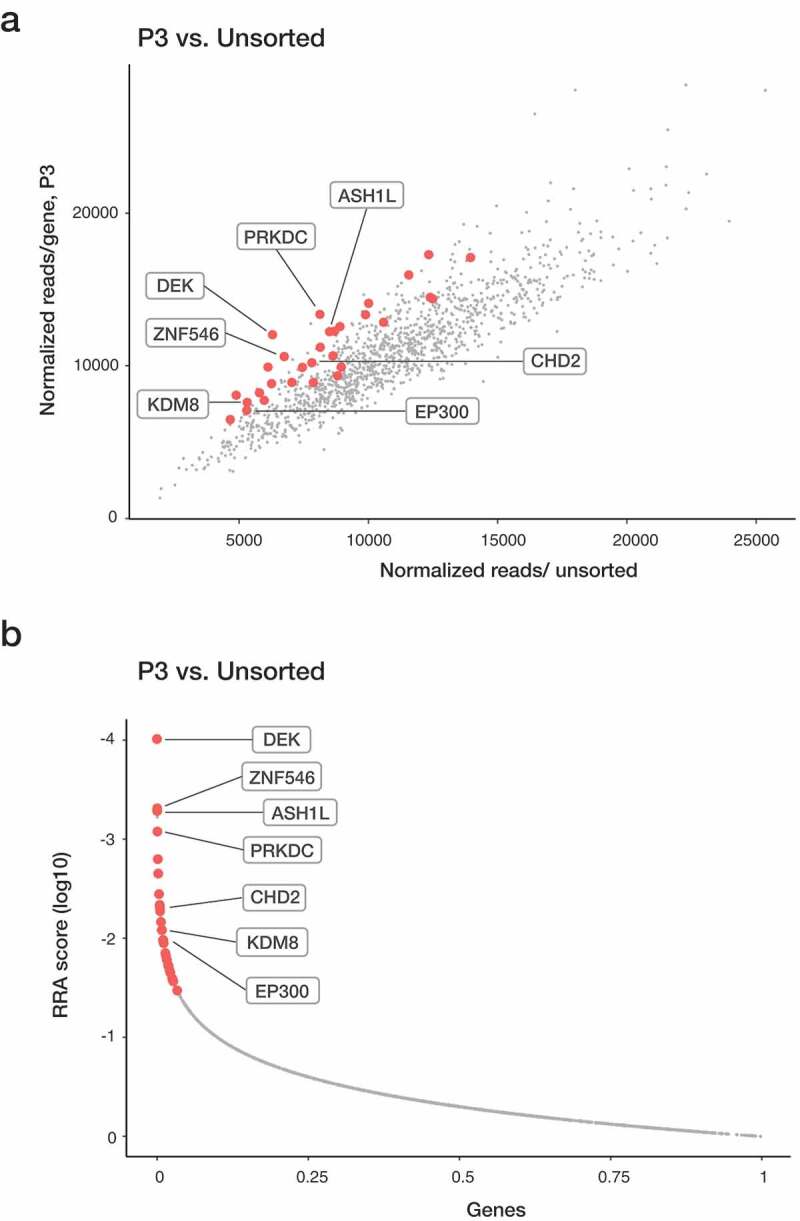
Enriched genes in the P3 population from the CRISPR-Cas9 screen. Sequencing read counts for guides targeting each gene in P3 vs unsorted cells. Replicate overlap of the top 10% enriched genes (in red) and specific genes of interest are highlighted. (a) Genes significantly enriched after 72-h treatment with PMA were identified with MAGeCK program. A modified robust ranking aggregation (RRA) algorithm was used to rank sgRNAs based on p-values.

Guides targeting ribosomal proteins showed depletion in unsorted cell populations, demonstrating that CRISPR-Cas9 editing worked in our K-562 cell line model. To be considered enriched, genes were required to be among the top 10% enriched in a given sorted population relative to unsorted PMA treated cells (by RRA) and have a >0.2 mean log fold change (average of all four guides). Only genes that met these criteria in both biological replicates were considered candidate genes. The duplicates for each cell population significantly overlapped, demonstrating reproducibility within the experimental set up. In P1, 14 candidate genes were enriched in both replicates (P = 0.0072). In P2 and P3 population were 13 (P = 0.0047) and 30 (P = 1.58 × 10^−11^) candidate genes identified, respectively (Table S1). The candidate genes enriched in P1 after PMA induction are potential drivers of myeloid differentiation, while the candidate genes in P2 and P3 may inhibit differentiation, potentially by promoting proliferation. In addition to identifying novel epigenetic regulators for myeloid differentiation, our screen identified previously described myeloid regulators, demonstrating the relevance of the differentiation model system. The P1 population contains, for example, *ARID4B* (Figure S2 and Table S1), which have been shown to inhibit the cell cycle progression that is consistent with an indirect role in promoting differentiation [15,16]. Arid4b-deficient mice display a perturbed haematopoiesis in all haematopoietic blood lineages and with time develop myeloproliferative disorders and AML, indicating a role as positive regulator of myeloid differentiation [16]. The P3 population is enriched for guides targeting *KMT2A/MLL and ASH1L* (Figure 2 and Table S1). The *KMT2* family plays an essential role in haematopoiesis, where especially *KMT2A* has been demonstrated to be important for the development of AML [17]. Chromosomal translocation of the 3ʹ-end of the *KMT2A* gene leads to the rapid development of AML, despite unchanged expression levels of the *KMT2A* fusion gene [17]. ASH1-like (*ASH1L*) is expressed in haematopoietic stem and progenitor cells (HSPC) and has been suggested to regulate HSC self-renewal via transcriptional activation of Hox genes in mice [18]. Thus, several of the identified candidates have been shown to have regulatory roles in haematopoiesis, in line with the result of our screen, supporting that the novel identified candidates may play an important role in myelopoiesis (Figures 2, S2, S3 and Table S1).

All types of epigenetic regulators are among the novel candidates (Table S1). The sgRNAs for chromatin remodeller *CHD2* were among our enriched candidates in P3 (Table S1, Figures 2, S2 and S3). Since other CHD family members have been proposed to play a role in pluripotency and myeloid leukaemia [10,12], the function of *CHD2* in myeloid differentiation was further characterized.

### CHD2 inhibits megakaryocytic differentiation and promotes proliferation

**Figure 3.**
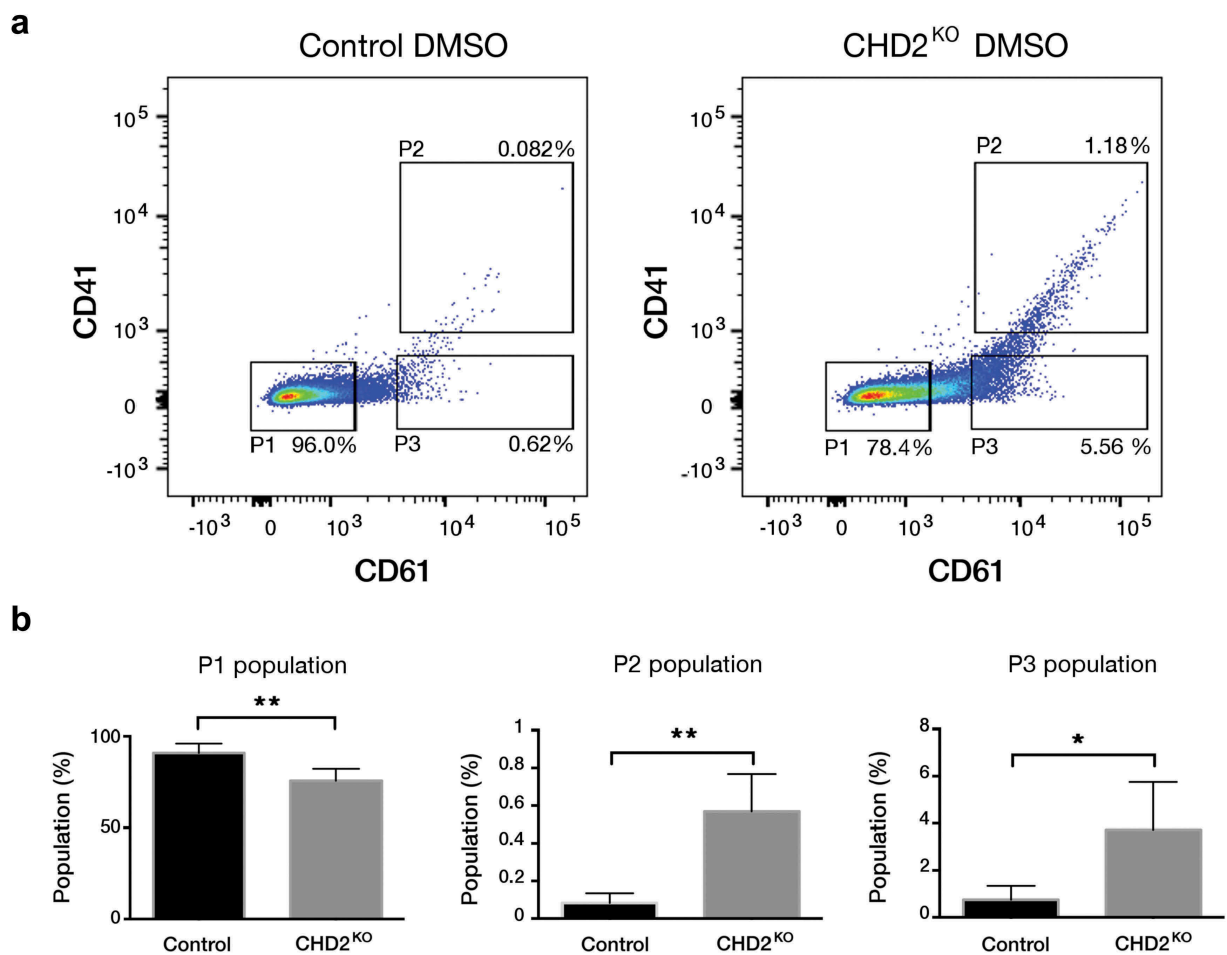
CHD2 KO causes megakaryocytic characteristics. (a) Representative flow cytometry chart for uninduced (DMSO) CHD2 KD versus control sample. The proportions of the cells for CD61 and CD41 were analysed with flow cytometry in three different populations (P1, P2 and P3). (b) Bar graphs show proportions of different cell populations for CD61 and CD41 markers for CHD2 KO versus Control samples. Data are represented as means ± SD. The p-values were calculated using an unpaired t-test with Welch’s correction (n = 5).

**Figure 4.**
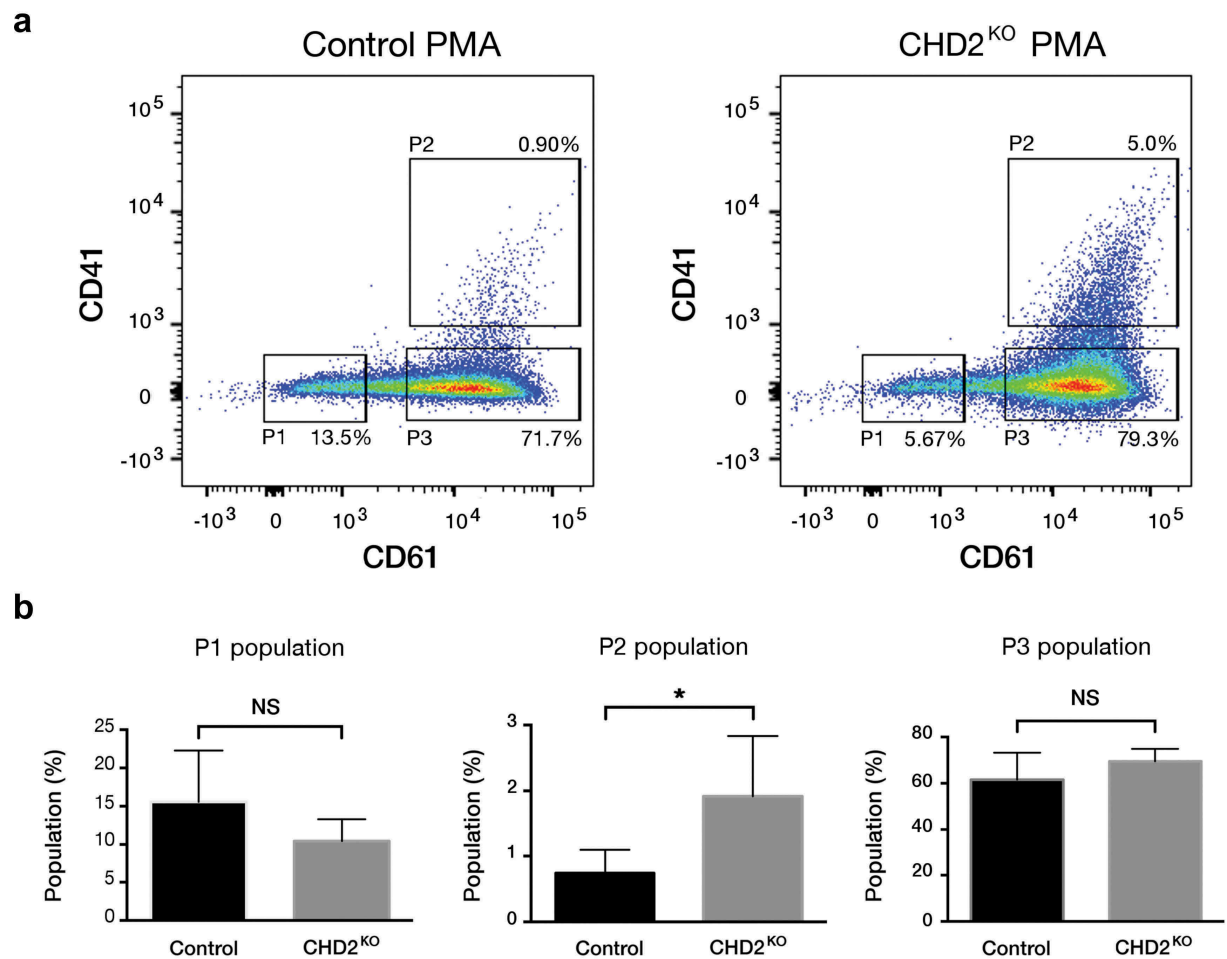
PMA increases megakaryocytic characteristics in CHD2 KO K-562 cells. (a) Representative flow cytometry chart for CHD2 KO versus control sample with PMA treatment (5 nM for 24 h) and the percentage of CD61 and CD41 surface markers in three different population: P1, P2, P3. (b) Bar graphs show proportions of different cell populations for CD61 and CD41 markers for CHD2 KO versus control samples. Data are represented as means ± SD. The p-values were calculated using an unpaired t-test with Welch’s correction (P = 0.0427 for P2) (n = 5).

To validate and further analyse the inhibitory role of CHD2 on megakaryocytic differentiation we changed to a CRISPR_Cpf1 system to minimize the risk for off-target effects. The K-562 cell line was transfected with 4 different sgRNAs targeting *CHD2* and cloned into a Cpf1 vector. Single-cell clones from transfected cells were expanded and the genomic editing of the *CHD2* locus was analysed by Sanger sequencing. All CHD2 knock out (KO) clones had at least one introduced indel in *CHD2* (Figure S4(a)) that will interrupt the reading frame and knock out CHD2. The clones were also tested for CHD1 levels by immunoblot to control for target specificity or possibly compensatory CHD1 expression. No changes of the CHD1 levels were detected in our clones compared to the controls (Figure S4(b)). To validate the differentiation phenotype from the screen we analysed the megakaryocytic differentiation after 24-h PMA/DMSO induction and analysed CD61/CD41 markers by flow cytometry. Analysis was performed after 24-h PMA treatment to detect early differentiation effects of PMA treatment. The CHD2 KO cells showed significantly enhanced spontaneous differentiation compared to the control cells in the DMSO samples (Figure 3(a,b)). The undifferentiated P1 population decreased from 96% to 78.4%, and the two differentiated P2 and P3 populations increased from 0.082% to 1.18% and 0.62% to 5.56%, respectively. In response to 24-h PMA treatment, we detect a significant increase in the double-positive (CD61+ CD41+) P2 population in the CHD2 KO cells compared with the control cells (Figure 4(a,b)).

**Figure 5.**
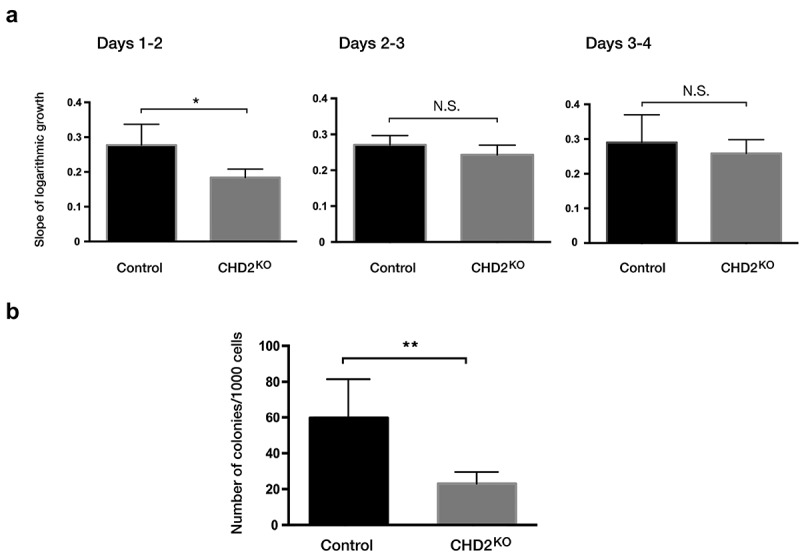
Proliferation rate is lower in CHD2 knocked down cells. (a) Bar chart for the slope of logarithmic growth cells for CHD2 KD cells compared to control cells for 3 interval days. Cells were counted using Trypan blue as dead cell exclusion marker with a viability of over 95% (n = 4). The slope of the growth between day 1 and day 2 has P = 0.0454 for which indicate the difference in growth between CHD2 KD and control cells are significant. (b) Bar chart for the number of colonies in CFU assay. Data are represented as means ± SD. The p-values were calculated using an unpaired t-test with Welch’s correction (n = 3).

To investigate whether the effect on differentiation is coupled to cell proliferation, cell growth was followed for 4 days. The live-cell population was counted with trypan blue staining for dead cell exclusion. The logarithmic-growth curve for CHD2 KO compare with controls (Figure S5) showed that CHD2 KO clones initially had a significantly lower proliferation rate compared to the control cells (Figure 5(a)). However, after day 2, the CHD2 KO cells showed similar growth rate as the controls (Figure 5(a)). This indicates that CHD2 plays a role in a role in growth at low cell density. Thus, to determine how CHD2 affects the ability of single cells to establish a visible colony, we evaluated the colony-forming capacity of *CHD2* KO clones. Consistent with the initial decreased growth, a colony-forming unit (CFU) assay showed that CHD2 KO clones had a significantly (p = 0.0079) reduced colony-forming capacity compared to control clones (Figure 5(b)).

### CHD2 binding promotes transcriptional activity

**Figure 6.**
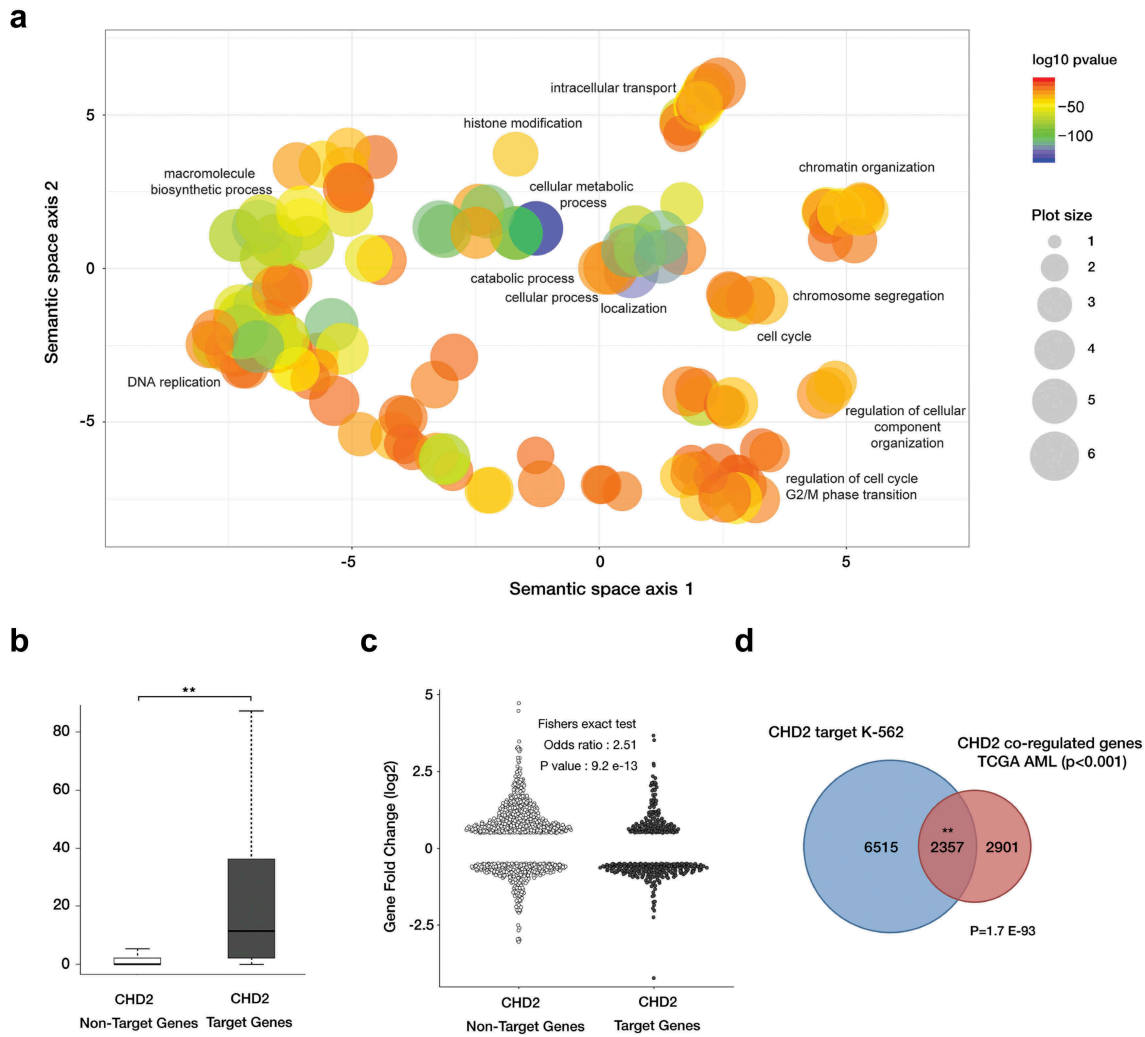
CHD2 target genes in K-562 are involved in a great variety of cellular processes and correlated with high expression levels. (a) CHD2-enriched gene ontology (GO) terms are visualized using REVIGO [37], which allows to cluster GO terms according to their similarity in a semantic space. GO terms with a p-value ≤0.001 are shown using ‘medium’ for the allowed semantic similarity. (b) Global expression levels (extracted from CAGE FANTOM data [20]) between CHD2 target genes and non-target genes in K-562. P-value <0.001. tpm, transcript per million. (c) RNA-sequencing analysis of CHD2 KO and control cells integrated with CHD2 ChIP-sequencing data from K-562 cells retrieved from the ENCODE project (ENCSR000EHD). (d) Venn diagram showing between the overlap between CHD2 target genes in K-562 cell line and CHD2 co-regulated genes in TCGA AML cohort.

Next, we sought to elucidate how CHD2 promotes differentiation. We have previously demonstrated that CHD2 is recruited in an RNA polymerase II-dependent manner to transcription start sites where it promotes nucleosome disassembly [19]. Analysis of CHD2 ChIP-sequencing data in the K-562 cell line retrieved from the ENCODE project (ENCSR000EHD) showed that CHD2 binds to over 30% of all annotated genes (8872 out of 27,113). Gene-ontology analysis of CHD2 target genes showed that CHD2 binds to genes involved in several fundamental cell functions, such as cell cycle process, DNA replication, chromatin organization and histone modification (Figure 6(a)). Comparing the global expression levels (retrieved from CAGE FANTOM 5 data set) [20] of CHD2 target genes with non-target genes in K-562 cells revealed that CHD2 target genes had significantly higher expression (Figure 6(b)), indicating that CHD2 positively regulates gene expression in a general manner in K-562 cells. Indeed, RNA-sequencing analysis showed that CHD2 target genes are down regulated in the CHD2 KO clones, demonstrating a role for CHD2 in active transcription (Figure 6(c)). CHD2 target genes are significantly (p9.2 E-13) more repressed than non-CHD2 target genes in CHD2 KD clones (Figure 6(c)).

To analyse the role of CHD2 in primary AML samples, we collected significant CHD2 co-expressed genes of 162 patients (p < 0.001) of clinically annotated adult cases of *de novo* AML from the Cancer Genome Atlas (TCGA) cohort [21] using Cbioportal [22,23]. To analyse whether the CHD2 co-expressed genes could also be direct CHD2 targets, the list of CDH2 co-expressed genes was compared to the list of genes with CHD2 binding in K-562 cells. Almost half (45%, Hypergeometric test P = 1.7 E-93) of the co-expressed genes in the TCGA AML cohort are also direct CHD2 targets (Figure 6(c)), suggesting that CHD2 promotes AML-specific transcription.

## Discussion

We have performed a CRISPR-Cas9 screen targeting 1092 epigenetic factors in a myeloid differentiation model to identify epigenetic factors with a role in myelopoiesis. Our screen yielded 57 epigenetic regulators with a potential regulatory role in myeloid differentiation. We chose to validate and characterize the role of the chromatin remodeller *CHD2*. Different family members of the CHD chromatin remodeller have been shown to play a role in development and tumorigenesis [10,12]. *CHD2* is a chromatin remodeller that contains tandem chromodomains in the N-terminal region, which recognize methylated histone tails and serve an autoinhibitory role for both DNA-binding and ATPase activities of CHD2 when not bound to methylated histones. The C-terminal part of CHD2 contains a putative SANT-SLIDE-like DNA-binding domain (DBD) that is important for ATPase and chromatin remodelling activities [24].

CHD2 has been shown to play an important role in the normal development of different tissues. Body weight in different developmental stages is significantly lower in Chd2−/− mice compared with wild type and heterozygous mice. Chd2−/− mice display growth delay and perinatal lethality, while heterozygous animals have reduced neonatal viability, shorter life span, and develop lymphomas [25,26]. In addition, CHD2 has been suggested to play an essential role in brain development and long-term memory [27,28]. In mouse embryos, Chd2 is essential for both neuron differentiation and self-renewal capacity [29]. This manifests clinically: *CHD2* disruption in human associates with epilepsy and mental deficiency [30] or scoliosis [31]. CHD2 is also essential for several developmental processes in humans. Small deletions of the CHD2 locus can cause a rare genetic disorder with growth retardation, cardiac defects and early-natal lethality [32,33].

In blood, histological studies on neonatal mouse livers have shown that Chd2 mutation can cause aberrant distribution in haematopoietic lineages, with a bias towards megakaryocytes in Chd2 mutant mice [25]. In addition, the majority of Chd2 heterozygous mutant mice die because of multiple lymphomas and lymphoid hyperplasia, supporting the role of CHD2 in haematologic malignancies [25,26]. Consistently, *CHD2* mutation is one of the most frequently reported genetic alterations in chronic lymphocytic leukaemia (CLL), indicating an additional role in lymphoid development [34].

In this study, we identified CHD2 as a regulator of megakaryocytic development and showed that CHD2 is essential for myeloid differentiation. CHD2 has also been suggested to participate in the regulation of other differentiation programs such as muscle cell differentiation [35]. In the differentiation of myoblasts into muscle cells, Chd2 interacts with the transcription factor MyoD and facilitates H3.3 deposition at myogenic loci, promoting differentiation [35]. Our analysis of ENCODE CHD2 ChIP-sequencing data shows that CHD2 have 8872 target genes, suggesting that the CHD2 interaction is not mediated by a specific transcription factor, such as MyoD in muscle differentiation, but rather is coupled to the Polymerase II machinery and active transcription of promoters and enhancers as previously suggested [2]. Indeed, RNA-sequencing analysis showed a general decrease in the expression of CHD2 target genes in CHD2 KO cells, which is consistent with a general role for CHD2 in transcriptional activation in myeloid cells.

We have previously shown that *CHD2* is ubiquitously expressed in haematopoiesis [2], which is in line with a general core function in transcriptional regulation in blood cells. Here we showed that CHD2 KO leads to spontaneous megakaryocytic differentiation that is further induced by PMA treatment. Notably, CHD2 appears to inhibit differentiation and promote cell growth in myeloid cells. Our finding confirms an important role for *CHD2* in human megakaryocytic differentiation. In addition to increased differentiation, we observed a reduced proliferation at low cell density and decreased ability to form colonies in CHD2 KO cells.

In conclusion, we have identified several novel potential epigenetic regulators for myeloid differentiation, using a CRISPR-Cas9 screen in a myeloid differentiation model. We have validated and characterized a role for CHD2 in megakaryocytic differentiation and cell growth.

## Materials and methods

### Cell culture

Low passage K-562 cells were cultured in Iscove’s Modified Dulbecco’s Medium (IMDM) (12,440,061, ThermoFisher Scientific) supplemented with 10% foetal bovine serum (10,270,106, ThermoFisher Scientific).

### PMA differentiation

Phorbol 12-myristate 13-acetate (PMA) (P8139, Sigma-Aldrich) (5 nM) or dimethyl sulphoxide (DMSO) (D4540, Sigma-Aldrich) as vehicle were added to low passage K-562 cells for 1 or 3 days and megakaryocytic differentiation was measured by light microscopy, CD61 expression levels by qPCR and also flow cytometry analysis for CD61 (PE Mouse Anti-Human CD61, 555754, BD biosciences) and CD41 (APC Mouse Anti-Human CD41a, 559777, BD biosciences) [13,36].

### CRISPR-Cas9 screen

The human myeloid leukaemia line K-562 (ATCC® CCL-243™) was first made to stably express the Cas9 nuclease as described in [14]. In brief, a construct coding for Cas9, blasticidin resistance, and a single guide against HPRT1 were lentivirally introduced. This allows sequential selection for functional Cas9 with blasticidin (A1113903, ThermoFisher) and 6-thioguanine, a nucleotide analog lethal in HPRT1+ cells. Cas9 expression was confirmed by western blot.

We designed a 5048-guide Chromatin-UMI CRISPR guide library targeting 1092 epigenetic regulators, 5 selected transcription factors and 35 ribosomal proteins as positive control with four guides per gene, plus 200 non-targeting control guides. In addition, guides against 80 olfactory receptor genes, which should cut but not affect differentiation, were added as negative controls. All guide sequences were from the genome-wide Brunello sgRNA library [37]. We selected chromatin factor genes from two sources: the Epifactors Database of confirmed chromatin actors [38], plus additional putative chromatin actors selected based on protein domains [39]. Guides were then cloned in pool (oligos synthesized by CustomArray) to include Unique Molecular Identifiers [14] and packaged into lentivirus.

Functional titre of this virus was determined for K-562 cells by serial dilution of the virus followed by puromycin selection. Cas9-expressing K-562 cells were transduced in duplicate with this Chromatin-UMI library at an approximate MOI (multiplicity of infection) of 0.4 and 2000 cells/guide) in 10 µg/ml polybrene. Transduced cells were selected with puromycin (4 µg/ml) from post-transduction day two to four, and then split into two replicates.

Genomic DNA was isolated using the QIAamp DNA Blood Maxi kit (51,192, Qiagen), and guide and UMI sequences were amplified by PCR [14]. NGS data were analysed with the MaGeCK software [40] and by UMI lineage dropout analysis [14].

### ChIP-sequencing data analysis

ChIP-sequencing CHD2 peaks coordinates (GRCh37/hg19) in K-562 cell line were downloaded from ENCODE project. A human reference gene list using NCBI RefSeq track and GRCh37/hg19 assembly was extracted from Table Browser function in UCSC website. The genomic coordinates of the reference genes list (including −500bp TSS promoter region) were intersected with the genomic coordinates of ENCODE CHD2 peaks using bedtools intersection function (https://bedtools.readthedocs.io/en/latest/content/tools/intersect.html).

Then, a gene ontology (GO) analysis was run for the unique gene names associated to CHD2 peaks, using as background all genes in the reference gene list. Enriched GO terms were extracted using GORILLA online tool [41] and visualized with REVIGO [42], using the following parameters: ‘Medium’ for the allowed similarity and ‘SimRel’ for semantic similarity measure.

### TCGA data

Co-expressed genes with CHD2 expression were analysed from adult cases of *de novo* acute myeloid leukaemia cohort [21] using Cbioportal [22,23].

### Cloning gRNA and transfection

The CRISPR_Cpf1 targeted sites for our targets were evaluated and selected using the web tool Benchling. Four selected sgRNA cloned to the vector pY095 (pY095 was a gift from Feng Zhang (Addgene plasmid # 84744; http://n2t.net/addgene: 84744; RRID:Addgene_84744)) [43]. The cloned vector and the original vector as control were transfected into low passage K-562 cells with Lonza Nucleofector device (V4XC-2012, Lonza).

### Single cell colonies sorting

K-562 cells sorted 72 h after transfection for GFP positive and top 10% of the positive cells collected in 96 wells plate for single cell sorting (BD FACSAria™ IIu flow cytometer).

### RNA-sequencing and analysis

Total RNA was subjected to quality control with Agilent Tapestation according to the manufacturer’s instructions. To construct libraries suitable for Illumina sequencing the Illumina TruSeq Stranded mRNA Sample preparation protocol which includes mRNA isolation, cDNA synthesis, ligation of adapters and amplification of indexed libraries were used. The yield and quality of the amplified libraries were analysed using Qubit by Thermo Fisher and the Agilent Tapestation. The indexed cDNA libraries were normalized and combined and the pools were sequenced on the Illumina Nextseq 550 for a 75-cycle v2.5 sequencing run generating 75 bp single-end reads. Basecalling and demultiplexing were performed using CASAVA software with default settings generating Fastq files for further downstream mapping and analysis. Bcl files were converted and demultiplexed to fastq using the bcl2fastq program. STAR was used to index the human reference genome (hg38/GRCh38) and align the resulting fastq files. Mapped reads were then counted in annotated exons using featureCounts. The entrez gene annotations and reference genome were obtained from UCSC. The count table from featureCounts was imported into R/Bioconductor and gene counts were normalized using the EdgeR package and TMM normalization.

Plotting was performed in R using the ggplot2 and ggbeeswarm packages. Gene log2 fold change values obtained from edgeR were used for the plotting. Only genes that had a log2 fold change of ± 0.5 were plotted. The odds ratio and p value were obtained using the Fisher’s exact test in R applying it to genes separated into four groups, up or downregulated and whether they had a CHD2 peak or not.

### Sanger sequencing

The genomic DNA was purified with PureLink Genomic DNA Mini Kit (K1820-01, Sigma-Aldrich). Primers were designed using primer-BLAST to amplify the four exon regions that are targeted by the guide RNA. The PCR products were purified with QIAquick Gel Extraction Kit (28706, Qiagen) and sequenced in Eurofins Genomics.

The following primers were used: Forward Primer Exon 3: GCCTCTGAAGAAGCTTCGGG, Reverse Primer Exon 3: GTCTGCTAGCTTGTCCTAGGCT, Forward Primer Exon 7: CTATCATGAACAACATGCTGTG, Reverse Primer Exon 7: TGAAGATAATGCCACTAAGTCACC, Forward Primer Exon 14: GCATTGGTGTCGTTGCTGTT, Reverse Primer Exon 14: ACACAGATTCTAACCCAGCTCT, Forward Primer Exon 28: TCACCTGCTGCCACTAACAG, Reverse Primer Exon 28: CCAGGTTGCGAAGACTCACT.

### Western blot

For western blotting, samples were lysed using RIPA buffer with proteinase inhibitor cocktail (11873580001, Sigma-Aldrich). Equal amounts of cell lysate were run on precast gels (456-1045, 456-1024, Bio-Rad) and transferred to PVDF membranes (1704156, Bio-Rad). The membranes were blocked with 5% milk/TBS-T for 1 h at room temperature. Membranes were incubated overnight with primary antibodies at 4°C. Membranes were washed and incubated for 1 h at room temperature with the corresponding secondary antibodies followed by washing steps. The bands were detected with 1:1 ratio of SuperSignal West Femto Maximum Sensitivity Substrate (34096, ThermoFisher Scientific) with ChemiDoc Touch imaging system, BIO RAD. CHD1 antibody NB100-60411, Novus Biological, HSP 90α/β antibody (H-114): sc-7947 (Santa Cruz Biotechnology).

### Cell growth assay

Cells were seeded at a concentration of 70,000 cells per ml and cell growth was measured every 24 h with the Automated Cell Counter TC20, BIO RAD using Trypan Blue as dead cell exclusion method.

### Colony-forming unit (CFU) assay

For each group, 1000 K-562 cells in IMDM (Iscove’s modified Dulbecco’s media) with 2% FBS were seeded into methylcellulose medium in the presence of cytokines (MethoCult™ H4034 Optimum, StemCell Technologies). Colonies were counted after 11 days of incubation at 37ºC [44,45].

## Supplementary Material

Supplemental Material
