# Supplementary material for: A regulatory role for CHD2 in myelopoiesis
Source: Epigenetics. 2020 Jan 10;15(6-7):702–14. doi: 10.1080/15592294.2019.1710913 (PMC7574388; doi:10.1080/15592294.2019.1710913)
Supplement: Supplemental Material [file KEPI_A_1710913_SM8001.pdf]

Supplementary data

A regulatory role for CHD2 in myelopoiesis

Farzaneh Shahin Varnoosfaderani, Anna Palau, Wenbo Dong, Jenna Persson, Mickael Durand-Dubief, J Peter Svensson and Andreas Lennartsson.

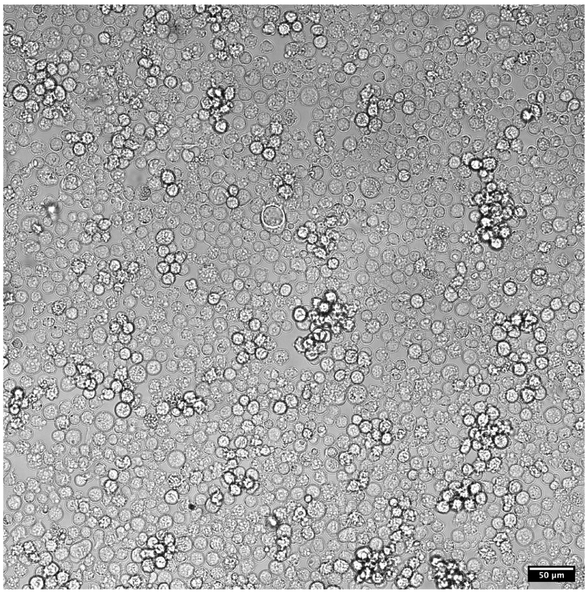

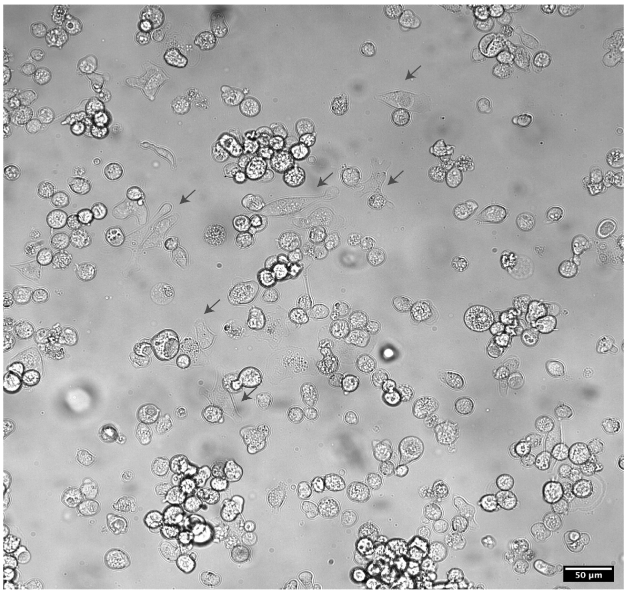

DMSO 72 H **PMA 72 H**

Supplementary Figure S1. Megakaryocytic differentiation with PMA treatment. CRISPR-Cas9-K-562 cells were incubated with DMSO/ PMA (5 nM for 72 hours) and morphological changes were observed with microscope. Scale bars represents 50 μm.

Supplementary Figure S2

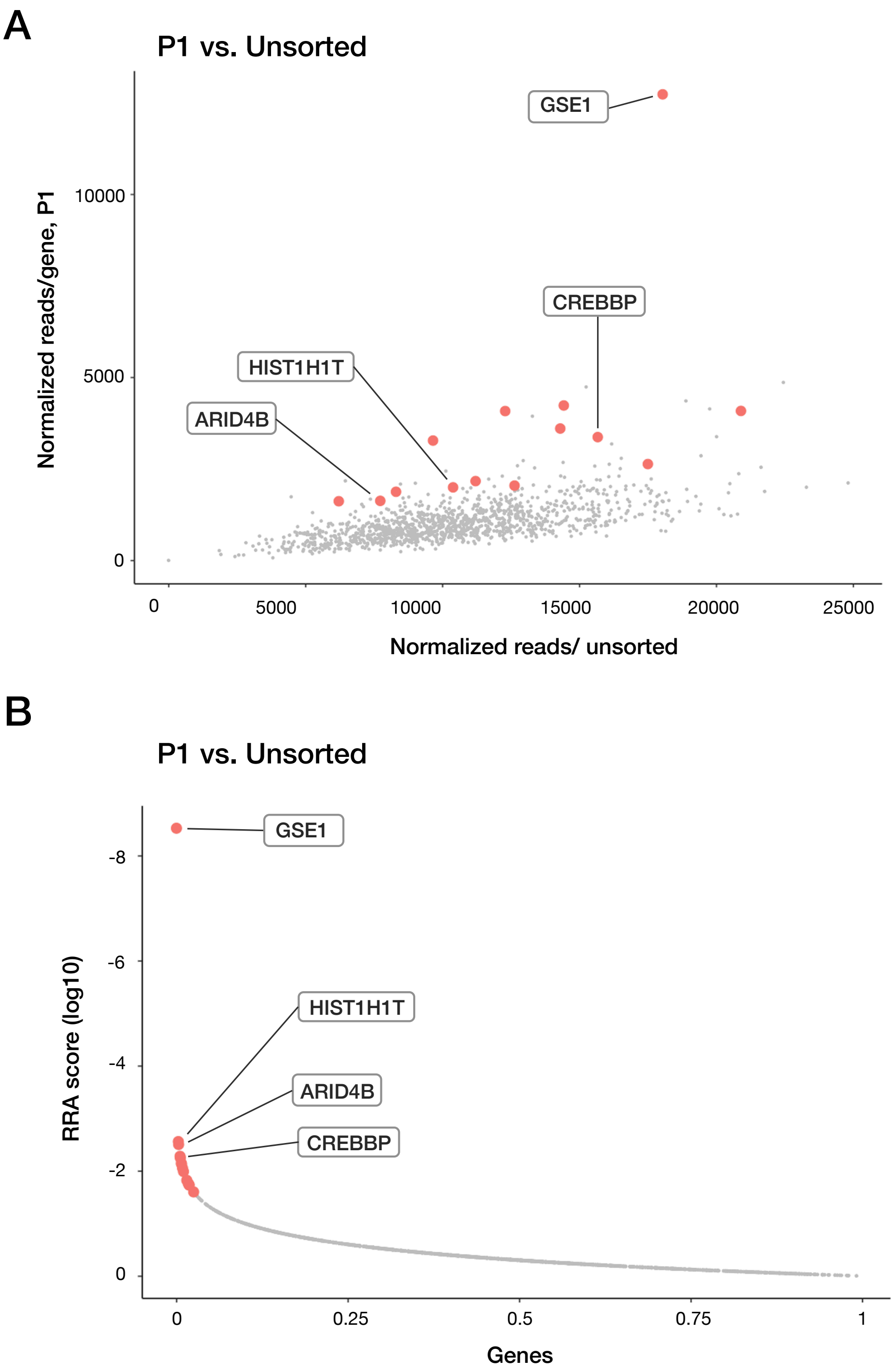

Supplementary Figure S2. Enriched genes in the P1 population from the CRISPR-Cas9 screen (A) Sequencing read counts for guides targeting each gene in P1 vs unsorted cells. Replicate overlap of the top 10% enriched genes (red) and specific genes of interest are highlighted **(B)** Genes significantly enriched after 72 hours treatment with PMA were identified with MAGeCK program. A modified robust ranking aggregation (RRA) algorithm was used to rank sgRNAs based on p-values.

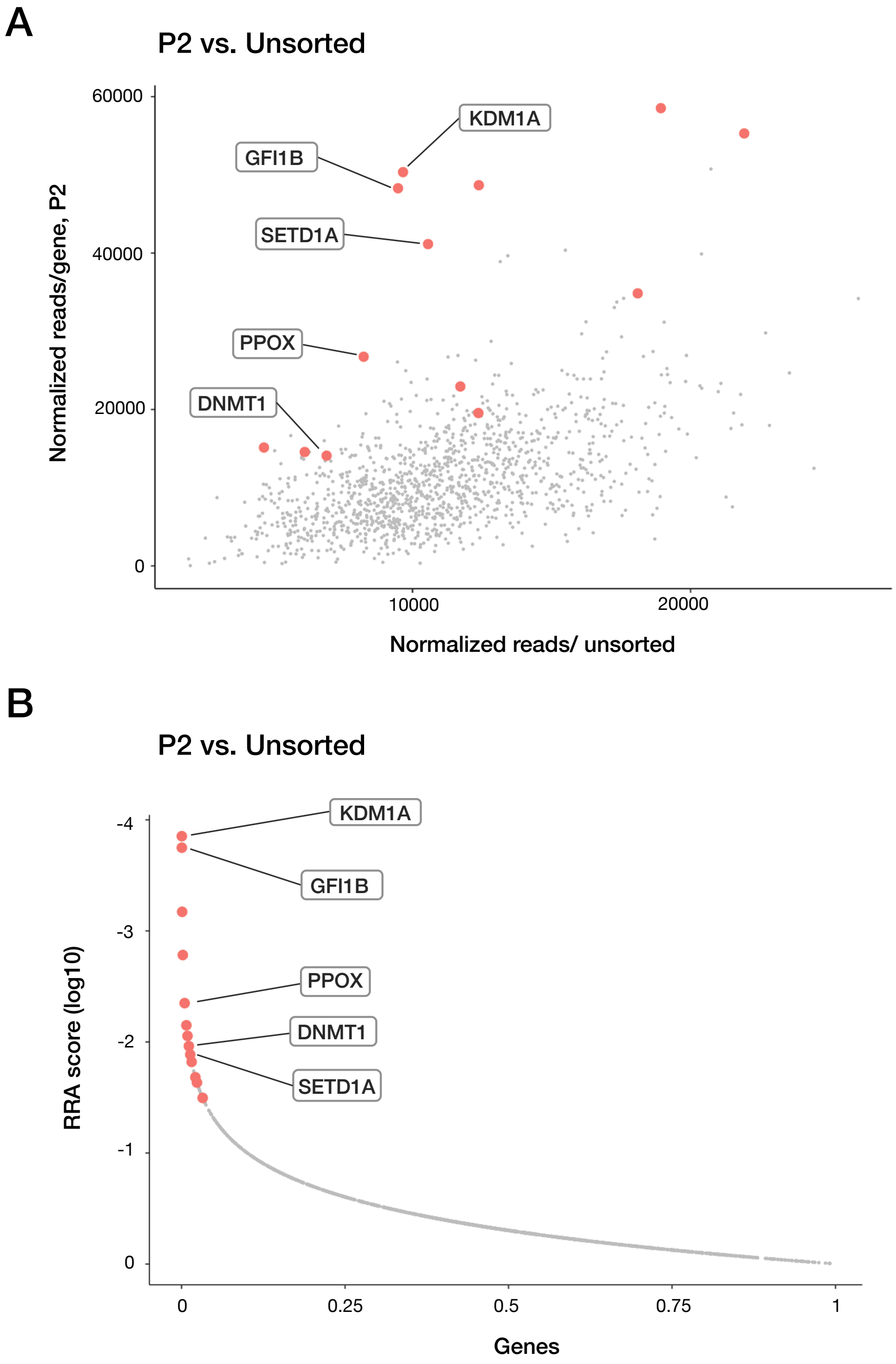

Supplementary Figure S3. Enriched genes in the P2 population from the CRISPR-Cas9 screen (A) Sequencing read counts for guides targeting each gene in P2 vs unsorted cells. Replicate overlap of the top 10% enriched genes (red) and specific genes of interest are highlighted **(B)** Genes significantly enriched after 72 hours treatment with PMA were identified with MAGeCK program. A modified robust ranking aggregation (RRA) algorithm was used to rank sgRNAs based on p-values.

A

Ctrl-Exon 3	5' - TTCCGACTGACTGCCTGAGTCTGAACCCGAA -3'
CHD2 KO - 1	5' - TTCCGACTGACTGCCTGAGTCTGAACCCGAA -3'
CHD2 KO - 2	5' - TTCCGACT--CTGCCTGAGTCTGAACCCGAA -3'
CHD2 KO - 3	5' - TTCCGACT--CTGCCTGAGTCTGAACCCGAA -3'
CHD2 KO - 4	5' - TTCCGACTGACTGCCTGAGTCTGAACCCGAA -3'
Ctrl-Exon 7	5' - TCCC--AAAAGGCAGACTC---GTCGAAG-AGCGGCT—AA - 3'
CHD2 KO - 1	5' - TCCCCAAAAAGGCAGACTCCGTCCCGAAGAAGCGGCTAAAA - 3'
CHD2 KO - 2	5' - TCCC--AAAAGGCAGACTC---GTCGAAG-AGCGGCT—AA - 3'
CHD2 KO - 3	5' - TCCC--AAAAGGCAGACTC---GTCGAAG-AGCGGCT—AA - 3'
CHD2 KO - 4	5' - TCCC--AAAAGGCAGACTC---GTCGAAG-AGCGGCT—AA - 3'
Ctrl-Exon 14	5' - TTATTTCTGCAGAAAGAAAAA - 3'
CHD2 KO - 1	5' - TTATTTCTGCAGAAAGAAAAA - 3'
CHD2 KO - 2	5' - TTATTTCTGCAGAAAGAAAAA - 3'
CHD2 KO - 3	5' - TTATTTCTGCAGAAAGAAAAA - 3'
CHD2 KO - 4	5' - TTATTTCTTCCAAGCAGAAAGAAAA - 3'
Ctrl-Exon 28	5' - TAAAGCCAACTTCGCTTACCCTCGCT - 3'
CHD2 KO - 1	5' - TAAAGGCCAACTTCGCCTTACCCTCGCT - 3'
CHD2 KO - 2	5' - TAAAGCCAACTTCGCTTACCCTCGCT - 3'
CHD2 KO - 3	5' - TAAAGCCAACTTCGCTTACCCTCGCT - 3'
CHD2 KO - 4	5' - TAAAGCCAACTTCGCTTACCCTCGCT - 3'

B

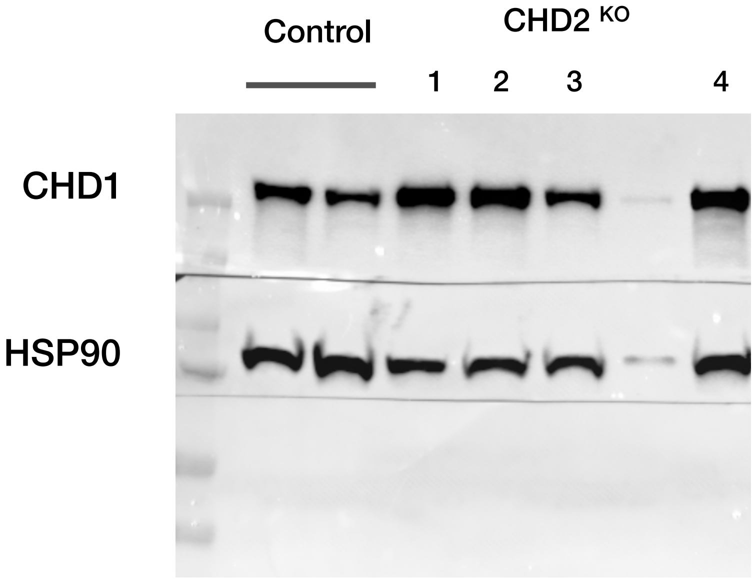

Supplementary Figure S4. Analysis of introduced indels in CHD2
(A) Sanger sequencing validation of the introduced CHD2 indels (in red) in the CHD2 KO clones. **(B)** Western blots for CHD1 in K-562 CHD2^{KO} clones and control cells. Anti-HSP 90α/β antibodies were used as loading controls.

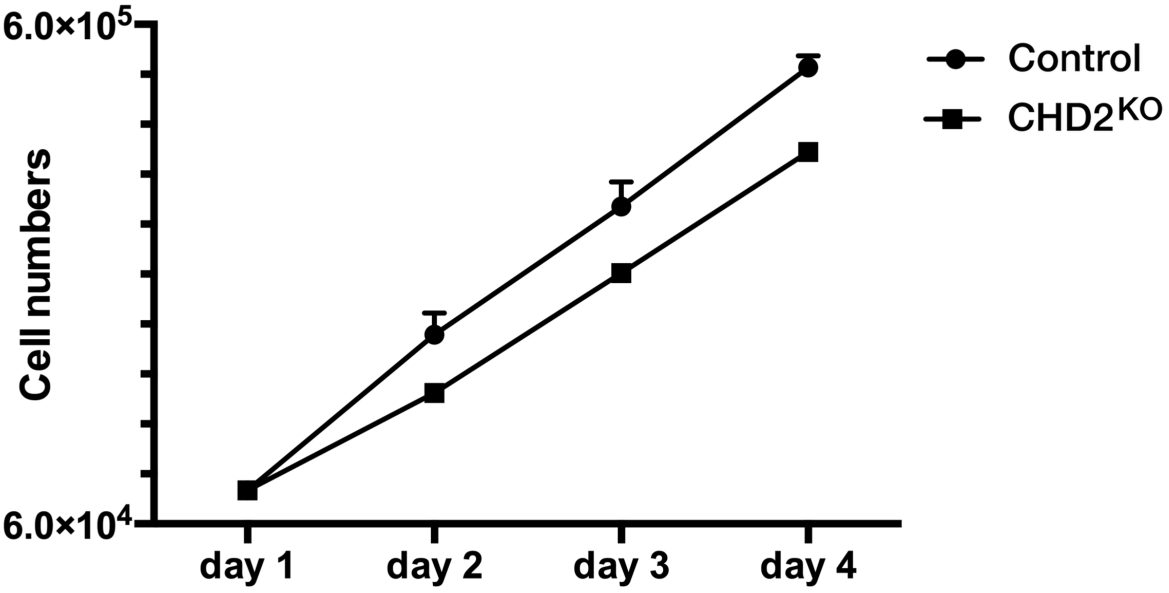

Supplementary Figure S5. Growth curve shows delay in CHD2 ^{KO} K-562 cells.
The X axis points are the days that cells counted after seeding in a 6-wells plate and on the Y axis is the cell numbers in logarithmic for growth cells for CHD2 knocked-out cells compared to control cells for 4 days. Cells were counted using Trypan blue as dead cell exclusion marker with a viability over 95% (n=4).
